# Comparative *in silico* analysis of EST-SSRs in angiosperm and gymnosperm tree genera

**DOI:** 10.1186/s12870-014-0220-8

**Published:** 2014-08-21

**Authors:** Sonali Sachin Ranade, Yao-Cheng Lin, Andrea Zuccolo, Yves Van de Peer, María del Rosario García-Gil

**Affiliations:** Umeå Plant Science Centre (UPSC), Department of Forest Genetics and Plant Physiology, Swedish University of Agricultural Sciences, SE-901-83 Umeå, Sweden; Department of Plant Systems Biology (VIB) and Department of Plant Biotechnology and Bioinformatics, Ghent University, Technologiepark 927, 9052 Ghent, Belgium; Istituto di Genomica Applicata, Via J. Linussio 51, 33100 Udine, Italy; Institute of Life Sciences, Scuola Superiore Sant’Anna, 56127 Pisa, Italy; Genomics Research Institute, University of Pretoria, Hatfield Campus, Pretoria, 0028 South Africa

**Keywords:** Angiosperms, Gymnosperms, Expressed sequence tags, Simple sequence repeats (SSR), Microsatellites

## Abstract

**Background:**

Simple Sequence Repeats (SSRs) derived from Expressed Sequence Tags (ESTs) belong to the expressed fraction of the genome and are important for gene regulation, recombination, DNA replication, cell cycle and mismatch repair. Here, we present a comparative analysis of the SSR motif distribution in the 5′UTR, ORF and 3′UTR fractions of ESTs across selected genera of woody trees representing gymnosperms (17 species from seven genera) and angiosperms (40 species from eight genera).

**Results:**

Our analysis supports a modest contribution of EST-SSR length to genome size in gymnosperms, while EST-SSR density was not associated with genome size in neither angiosperms nor gymnosperms. Multiple factors seem to have contributed to the lower abundance of EST-SSRs in gymnosperms that has resulted in a non-linear relationship with genome size diversity. The AG/CT motif was found to be the most abundant in SSRs of both angiosperms and gymnosperms, with a relative increase in AT/AT in the latter. Our data also reveals a higher abundance of hexamers across the gymnosperm genera.

**Conclusions:**

Our analysis provides the foundation for future comparative studies at the species level to unravel the evolutionary processes that control the SSR genesis and divergence between angiosperm and gymnosperm tree species.

**Electronic supplementary material:**

The online version of this article (doi:10.1186/s12870-014-0220-8) contains supplementary material, which is available to authorized users.

## Background

Microsatellites, also called SSRs (simple sequence repeats) or STRs (short tandem repeats), are 1-6 bp tandem repeat motifs present in both the coding and non-coding fractions of eukaryotic and prokaryotic genomes [1-3]. SSRs are especially abundant in transcribed regions of the genome making them a valuable molecular marker for genetic studies in plants [4]. SSRs result from mutations due to DNA-polymerase slippage during replication and unequal recombination [5]. SSRs are widely used in plant genetic research because of their co-dominant inheritance, relative abundance, multi-allelic nature, high reproducibility and ease of detection [6].

Expressed sequence tags (ESTs) are segments of expressed genes generated by single-pass sequencing of cDNA libraries [7]. In contrast to the genomic SSRs, EST-SSRs represent functional markers located in the coding fractions of the genome and changes in EST-SSRs length can cause a phenotypic effect, irrespective of the mutation site, whether it occurs in 5′- or 3′-UnTranslated Regions (UTRs) or in the Open Reading Frames (ORFs) [8]. The significance of EST-SSRs as a molecular tool in population genetic studies has been known for long [9]. In woody trees, EST-SSRs have been applied in population studies and analysis of genetic diversity in *Cycas* [10], *Picea* [11,12], *Prunus* [13,14], *Eucalyptus* [15,16] and *Populus* [17]; in hybrid selection in e.g., *Citrus* [18]; and also in genetic mapping in *Citrus* [19], *Quercus* [20,21] and *Pinus* [22]. Furthermore, unlike the genomic SSRs, EST-SSRs are easily transferable across species [23], therefore allowing studying polymorphism and genetic diversity in related species [9]. However, EST-SSRs have some disadvantages over genomic SSRs as EST-SSRs are known to be less variable than the genomic SSRs [24] and the amplicon size can also differ from the predicted size due to the effect of presence of introns in the flanking fractions [25].

With the advent of genomics, the availability of ESTs in the public databases, such as NCBI’s dbEST, has increased exponentially allowing for the identification of large numbers of EST-SSRs. For example, characterisation and comparative analysis of EST microsatellites in woody trees have been carried out in *Citrus* [26-28], *Betula* [29], *Fagus* [30], *Prunus* [31], *Quercus* [20], *Populus* [17,32], *Eucalyptus* [33-35], *Cryptomeria* [36,37], *Cycas* [38-40], *Ginkgo* [41], *Picea* [5,12] and *Pinus* [5,42]. However, analysis of SSRs for each individual EST genomic fraction (i.e., 5′- and 3′-UTR, and ORF) has only been carried out in *Quercus* [20], *Cryptomeria* [37] and *Pinus* [43]. Unfortunately, most of the results in those three studies are presented for the entire EST, which can lead to inaccurate results. For example, in *Cryptomeria* dimers are the most common motif in the 3′UTR fraction; moreover, when all three EST fractions are considered together, trimers are concluded to be the most frequent motif across the entire EST [37]. Furthermore, AT was shown to be the most frequent dimer motif as an overall result, whereas analysis of each EST fraction separately revealed AG as the most frequent dimer in the ORF fraction [37]. These results demonstrate that SSR characterization on the whole EST sequence as a unit will provide only partial information, which may be misleading and result in discrepancies across studies.

Other discrepancies in EST-SSRs motif abundance and distribution across different plant studies can be attributed to the parameter setup [25], annotation deficiency [44], and the selected EST-SSR analysis algorithm [20]. For example, higher abundance of EST-SSR dimers was reported in *Pinus* [45,46], whereas Yan et al. [47] reported trimers as the most abundant in the same genus. Thus, comparative EST-SSRs studies will be more reliable when the EST data sets are analysed by applying the same bioinformatics procedure. In this study, we performed a comparative analysis of SSRs in each genomic fraction of EST separately (5′UTR, ORF and 3′UTR), across selected angiosperm and gymnosperm genera with a focus on woody trees. The aim was to present highly comparable data on SSR-EST abundance, composition and distribution; for genomes that diverged ~350 Myr [48].

## Results

Table 1 shows values for EST-SSRs length and EST-SSR counts per genus across the 5′UTR, ORF and 3′UTR fractions (see also Additional file 1: Table S1).

**Table 1:** EST-SSR Counts per Mbp in each genomic fraction in: (a) Angiosperms and (b) Gymnosperms

**(a)**		**5′UTR**		**ORF**		**3′UTR**	
**Genus**	**Mean Genome size (pg)**	Motif length* (bp)	**Counts Mbp**	Motif length* (bp)	**Counts Mpb**	Motif length* (bp)	**Counts Mbp**
*Populus*	0.52	24.8 (6.04)	1483	25.7 (8.10)	580	24.8 (7.60)	653
*Eucalyptus*	0.6	25.5 (5.31)	2267	25.1 (5.48)	1248	25.3 (5.83)	638
*Betula*	0.62	23.1 (3.34)	1404	22.7 (3.02)	893	21.4 (1.51)	945
*Fagus*	0.56	24 (5.36)	1698	25.2 (7.01)	465	23.9 (4.90)	622
*Quercus*	0.87	24.2 (5.27)	2739	25.2 (7.98)	949	24.3 (6.78)	1109
*Citrus*	0.44	24.7 (6.75)	503	25.2 (8.15)	247	24.6 (6.87)	210
*Prunus*	0.57	27.5 (8.95)	7965	29.5 (11.38)	3089	26.9 (8.57)	4537
*Fraxinus*	0.93	24.2 (3.38)	551	28.7 (10.23)	183	22.4 (4.17)	236
**(b)**		**5′UTR**		**ORF**		**3′UTR**	
**Genus**	**Mean Genome size (pg)**	Motif Length* (bp)	**Counts Mbp**	Motif Length* (bp)	**Counts Mbp**	Motif Length* (bp)	**Counts Mbp**
*Picea*	18.1	29.7 (19.49)	247	32.1 (23.20)	206	28.6 (13.59)	250
*Pinus*	26.4	30.2 (17.80)	216	32.4 (19.09)	184	27.4 (11.98)	187
*Cryptomeria*	11.2	22.8 (3.95)	223	26.2 (10.37)	218	24.4 (8.40)	240
*Gnetum*	3.4	23.5 (4.22)	632	24.8 (7.96)	664	22.7 (3.64)	549
*Cycas*	14.7	23.8 (6.34)	173	26.4 (11.59)	109	24.9 (7.05)	399
*Zamia*	17	25.8 (6.55)	610	29.0 (12.64)	701	26.3 (8.4)	734
*Ginkgo*	11.8	24.5 (4.37)	386	29.2 (19.69)	210	27.1 (8.11)	539

*Standard deviation for EST-SSR length is in between parenthesis.

### EST-SSR length and complexity

There were no significant differences observed regarding EST-SSRs length between the three genomic fractions within and between taxa. In angiosperms, there was no significant association between genome size and EST-SSRs length for any of the EST fractions. In gymnosperms, however, there was a positive and significant association (r = 0.6; P-value < 0.03) between genome size and EST-SSRs motif length for all three EST fractions.

Perfect EST-SSRs were more frequent than compound ones in both taxa and in all three genomic fractions (Additional file 1: Table S2). In angiosperms, *Eucalyptus* (ORF) had the highest percentage of compound EST-SSR motifs (7.4%), while *Cycas* (3′UTR) had the highest percentage of compound SSR motifs (6.8%) in gymnosperms. None of the statistical tests made to compare proportions of complex EST-SSRs within and between taxa were significant. Furthermore, complexity was not significantly associated to genome size.

### EST-SSR abundance (motif counts per Mbp)

#### (i) Overall

In angiosperms, SSR counts showed a wide range across genera, with *Prunus* having an exceptional high abundance. EST-SSR counts were significantly higher in the 5′UTR fraction and lower in the ORFs. In gymnosperms, the SSR counts range was narrower than in angiosperms with *Zamia* and *Gnetum* having the highest values. EST-SSRs were significantly more abundant in the 3′UTR fraction, while there was a non-significant difference in abundance between the 5′UTR and ORF fractions. EST-SSRs were significantly more abundant in angiosperms than in gymnosperms. No association was found between density and genome size in any of the two taxa.

#### (ii) By motif size

The distribution of counts per Mbp for each of the EST-SSRs, according to motif size, is shown in Table 2. In angiosperms and gymnosperms, dimer motifs showed significantly higher number of counts in all three genomic fractions, followed by trimers, with the exception of *Citrus* (ORF, trimers > dimers), *Cryptomeria* (ORF, trimers > dimers) and *Gnetum* (5′UTR and ORF, trimers > dimers and trimers > hexamers, respectively). Non-significant differences between dimers and trimers were found in *Cryptomeria* (5′UTR) and *Gnetum* (3′UTR). In both taxa, the most frequent motif ranking in the ORF was dimer > trimer > hexamer. The same motif ranking was often observed in the UTRs in gymnosperms. Moreover, in angiosperms, hexamers are less often ranked in the third position in the UTRs, supporting a lower representation of hexamers in UTRs in angiosperms. Despite dimers being the motifs with higher number of counts in most of the genera across all three genomic fractions, the proportion of dimers to trimers was clearly lower in the ORF, indicating an enrichment of trimers in the ORF fraction in both taxa. Interestingly, *Gnetum* was the only genus where dimers rank third when it comes to abundance (ORF, trimers > hexamers > dimers); trimers and hexamers being relatively abundant across all three fractions. In *Fraxinus* and *Fagus*, trimers and hexamers were also rather abundant.

**Table 2:** Counts per Mbp of different SSR motifs in each genomic fraction in: (a) Angiosperms and (b) Gymnosperms

**(a)**	**Populus**			**Eucalyptus**			**Betula**			**Fagus**			**Quercus**			**Citrus**			**Prunus**			**Fraxinus**		
	**5′UTR**	**ORF**	**3′UTR**	**5′UTR**	**ORF**	**3′UTR**	**5′UTR**	**ORF**	**3′UTR**	**5′UTR**	**ORF**	**3′UTR**	**5′UTR**	**ORF**	**3′UTR**	**5′UTR**	**ORF**	**3′UTR**	**5′UTR**	**ORF**	**3′UTR**	**5′UTR**	**ORF**	**3′UTR**
Dimer	948	272	379	1821	699	459	1131	649	880	1304	230	397	2193	530	832	318	96	122	6854	2403	3568	522	124	143
Trimer	250	209	146	232	412	91	151	181	0	172	161	77	232	286	126	190	104	43	329	413	388	0	19	57
Tetramer	85	16	42	77	27	27	47	10	41	65	7	21	97	15	49	32	7	15	204	32	133	0	0	8
Pentamer	97	16	35	49	15	23	0	34	0	39	3	35	88	15	45	24	4	9	182	65	163	0	4	12
Hexamer	68	54	28	43	67	17	0	8	0	60	56	49	70	91	26	17	27	7	94	85	82	18	35	12
Heptamer	27	7	18	29	14	14	57	6	24	52	2	41	50	7	25	16	5	9	196	39	120	11	0	5
Octamer	6	2	2	2	4	5	0	0	0	3	1	0	3	1	3	4	1	2	67	18	49	0	0	0
Novamer	1	3	1	7	5	1	0	6	0	0	3	0	1	3	1	1	2	1	15	26	16	0	0	0
Decamer	3	2	2	7	6	2	19	0	0	4	2	1	5	1	2	2	2	2	23	7	18	0	0	0
**(b)**	**Picea**			**Pinus**			**Cryptomeria**			**Gnetum**			**Cycas**			**Zamia**			**Ginkgo**					
	**5′UTR**	**ORF**	**3′UTR**	**5′UTR**	**ORF**	**3′UTR**	**5′UTR**	**ORF**	**3′UTR**	**5′UTR**	**ORF**	**3′UTR**	**5′UTR**	**ORF**	**3′UTR**	**5′UTR**	**ORF**	**3′UTR**	**5′UTR**	**ORF**	**3′UTR**			
Dimer	183	128	199	169	121	140	46	58	116	133	104	182	118	84	354	503	504	578	319	164	483			
Trimer	14	41	12	5	30	8	43	85	47	260	355	169	10	13	12	35	143	60	52	24	12			
Tetramer	6	1	10	6	2	11	17	1	9	78	15	69	17	1	12	36	18	52	8	4	13			
Pentamer	24	5	12	12	4	8	41	8	20	45	31	41	9	1	6	11	9	21	7	1	17			
Hexamer	9	26	6	14	23	8	27	54	22	111	154	74	10	7	13	10	17	3	0	17	14			
Heptamer	8	1	8	7	2	6	37	8	22	0	2	5	7	2	2	15	8	19	0	0	0			
Octamer	2	1	1	1	1	1	8	1	1	0	0	0	0	0	0	0	0	0	0	0	0			
Novamer	1	3	1	1	2	2	0	3	0	0	2	0	0	0	0	0	1	0	0	0	0			
Decamer	1	1	1	1	1	2	4	1	2	5	0	8	0	0	0	0	2	0	0	0	0			

#### (iii) By dimer and trimer nucleotide composition

The counts for dimer and trimer nucleotide composition across genomic fractions and genera are shown in Table 3. In angiosperms, the AG/CT dimer motif showed the highest number of counts per Mbp in all genomic fractions and genera, followed by the AT/AT motif, with exception of *Betula* (AT/AT and AG/CT were present in similar numbers), *Citrus* (3′UTR; AT/AT) and *Populus* (3′UTR; AT/AT). In gymnosperms, AT/AT was the most abundant dimer motif in the 3′UTR fraction, with the exception of *Cryptomeria*, *Cycas* and *Gnetum* where AT/AT and AG/CT were present in similar numbers. In the 5′UTR and ORF fractions in gymnosperms, AG/CT was the most abundant motif in most of the genera, with the exception of *Cycas* (5′UTR), *Ginkgo* (ORF) and *Zamia* (ORF), where AT/AT and AG/CT were present in similar numbers; and *Ginkgo* (5′UTR), *Zamia* (5′UTR) and *Cycas* (ORF), where AT/AT was the most abundant. Overall, AT/AT was often the most abundant dimer in gymnosperms. The dimer motif CG/CG was absent in most of the genera and only present at low density in the ORF of *Populus* and *Quercus*.

**Table 3:** Counts per Mbp of dimer and trimer motifs in all three genomic fractions in: (a) Angiosperms and (b) Gymnosperms

**(a)**																								
**Motif**	***Populus***			***Eucalyptus***			***Betula***			***Fagus***			***Quercus***			***Citrus***			***Prunus***			***Fraxinus***		
	**5′UTR**	**ORF**	**3′UTR**	**5′UTR**	**ORF**	**3′UTR**	**5′UTR**	**ORF**	**3′UTR**	**5′UTR**	**ORF**	**3′UTR**	**5′UTR**	**ORF**	**3′UTR**	**5′UTR**	**ORF**	**3′UTR**	**5′UTR**	**ORF**	**3′UTR**	**5′UTR**	**ORF**	**3′UTR**
AC/GT	53	22	53	27	7	12	-	-	98	61	11	7	103	27	43	27	8	18	165	48	101	91	6	34
AG/CT	822	185	148	1788	684	431	1131	649	350	1173	181	262	1885	439	471	230	73	47	5992	2226	2655	431	113	109
AT/AT	73	57	178	7	8	15	-	-	432	69	38	128	205	63	317	60	16	57	697	129	811	-	6	-
CG/CG	-	8	-	-	-	-	-	-	-	-	-	-	-	1	-	-	-	-	-	-	-	-	-	-
ACG/CGT	27	42	14	29	63	11	-	-	-	6	10	-	5	18	1	6	17	2	15	63	9	-	-	-
ACT/AGT	23	23	14	17	4	9	66	34	-	13	24	5	27	38	29	6	9	4	29	114	136	-	4	21
AAC/GTT	10	12	9	5	1	-	85	57	-	15	30	10	39	55	17	3	10	1	40	43	22	-	-	-
AAG/CTT	93	46	43	98	63	28	-	77	-	111	52	22	125	91	31	38	28	12	168	90	86	-	4	11
AAT/ATT	30	11	51	-	5	3	-	14	-	13	11	36	24	14	42	29	16	21	34	26	41	-	4	25
ACC/GGT	26	35	7	-	25	3	-	57	-	-	12	5	6	45	3	5	10	1	7	33	5	-	4	-
AGG/CCT	33	32	7	26	63	12	-	-	-	13	22	-	5	20	3	2	7	1	36	33	26	-	-	-
CCG/CCG	4	7	1	52	183	12	-	-	-	-	1	-	1	6	-	2	6	1	-	5	-	-	-	-
**(b)**																								
**Motif**	***Picea***			***Pinus***			***Cryptomeria***			***Gnetum***			***Cycas***			***Zamia***			***Ginkgo***					
	**5′UTR**	**ORF**	**3′UTR**	**5′UTR**	**ORF**	**3′UTR**	**5′UTR**	**ORF**	**3′UTR**	**5′UTR**	**ORF**	**3′UTR**	**5′UTR**	**ORF**	**3′UTR**	**5′UTR**	**ORF**	**3′UTR**	**5′UTR**	**ORF**	**3′UTR**			
AC/GT	3	3	4	1	2	1	19	-	9	36	-	-	-	11	51	79	116	88	40	44	17			
AG/CT	95	93	37	101	80	40	19	46	55	76	60	91	54	33	143	170	194	182	120	60	120			
AT/AT	85	31	157	67	39	100	8	12	53	20	45	91	64	40	160	254	194	308	346	60	346			
CG/CG	-	-	-	-	-	-	-	-	-	-	-	-	-	-	-	-	-	-	-	-	-			
ACG/CGT	1	10	1	-	8	-	-	14	7	76	172	48	-	2	-	-	38	-	-	10	-			
ACT/AGT	1	1	2	-	3	-	9	8	7	38	31	-	-	-	-	-	14	11	-	-	-			
AAC/GTT	1	3	-	1	2	1	5	5	-	13	13	12	-	-	-	-	-	-	-	-	-			
AAG/CTT	1	6	2	1	7	-	6	26	10	76	57	86	10	9	-	8	32	16	-	5	-			
AAT/ATT	4	3	5	2	2	5	5	3	20	-	4	12	-	2	5	18	31	33	40	8	12			
ACC/GGT	1	2	-	1	2	-	11	-	-	27	-	-	-	-	-	-	9	-	-	-	-			
AGG/CCT	5	13	1	-	4	1	8	19	-	29	37	12	-	-	6	8	14	-	12	-	-			
CCG/CCG	1	4	-	-	2	-	-	10	4	-	14	-	-	-	-	-	-	-	-	-	-			

In the 3′UTR fraction in angiosperms and gymnosperms AAT/ATT was the most abundant trimer motif in all the genera with the exception of *Eucalyptus* (AAG/CTT, AGG/CTT and CCG/CCG were present in similar numbers), *Fraxinus* (AAT/AAT and ACT/AGT were present in similar numbers), *Prunus* (ACT/AGT most abundant) and *Gnetum* (AAG/CTT most abundant). In the 5′UTR and ORF fractions in angiosperms, AAG/CTT was the most abundant in all genera except in *Betula* (5′UTR; AAC/GTT and ACT/AGT were present in similar numbers), *Betula* (ORF; AAG/CTT, AAC/GTT and ACC/GGT were present in similar numbers), *Eucalyptus* (ORF; CCG/CCG most abundant), *Fraxinus* (ORF; AAG/CCT, ACT/AGT, AAT/ATT and ACC/GGT were present in similar numbers) and *Prunus* (ORF; ACT/AGT most abundant). Moreover, in the 5′UTR and ORF in gymnosperms, there was not a single trimer motif that ranked first, instead it varied across genera.

## Discussion

In this study we have investigated the occurrence of EST-SSRs in three EST genomic fractions (5′UTR, ORF and 3′UTR), in a genus-wise analysis in woody trees of two taxa, angiosperms and gymnosperms. Genus-wise EST-SSRs analysis for EST genomic fractions separately supports the unequal distribution of EST-SSR motifs across the EST sequences. EST-SSR length is positively associated with genome size in gymnosperms (i.e. larger genomes have longer EST-SSRs). However, EST-SSR density is not proportional to genome size; instead other factors seem to have contributed to the EST-SSR density in gymnosperms. We observed two main differences between angiosperm and gymnosperm genera, which may reflect evolutionary differences following their divergence 350 Myr [48], such as the increased presence of hexamers and AT-rich motifs in the gymnosperm genera.

### Low contribution of EST-SSRs to genome size diversity

Our EST-SSRs length values are in accordance with those previously reported in the literature [5,27,45]. In gymnosperms, we observe a positive and significant association between the EST-SSRs length and genome size. Thus, the largest genomes (*Pinus* and *Picea*) also have, on average, the longest EST-SSRs. Although this suggests a higher relaxation towards genome enlargement in those two genera, the yet small differences in length between the studied gymnosperm genera suggests that EST-SSRs length contribution to *Pinus* and *Picea* genome obesity may be only modest. Instead, EST-SSRs length has been suggested to be mainly the result of a balance between slippage events and point mutation [8], which have resulted in a rather homogeneous EST-SSRs length, as suggested before [45]. Unlike in gymnosperms, our analysis does not support an association between the EST-SSRs length and genome size in angiosperms. A potential association however could be masked by the multiple polyploidization events and their role in genome size diversification in angiosperms [49]. Although other factors may have played a role in genome size diversity in angiosperms; transposable element (TE) expansion seems to be the most determinant factor [50]. Conifer genome expansion can also be attributed to a large extent to TE expansion [51,52], although its role in genome size diversification is yet to be proven within the gymnosperm taxon.

Our values for percentage of perfect and compound EST-SSRs in *Gnetum* and *Pinus* agree with those reported by Victoria et al. [46] and are not correlated with genome size in any of the taxa. Our data also does not support the contribution of overall EST-SSRs abundance to genome size expansion. Instead, angiosperm genera with smaller genomes compared to those in gymnosperms show, on average a significantly higher abundance (four order of magnitude higher) of EST-SSRs. The lower density of EST-SSRs in gymnosperm compared to angiosperm species is in agreement with previous reports [5,45,47] and does not support a possible constant abundance of SSRs in the transcribed portions of the genome across species as suggested by Morgante et al. [4]. Several studies have concluded that EST-SSRs abundance is inversely related to the genome size [5,37], while others attribute EST-SSRs abundance partly to the action of selection and the effectiveness of mechanisms for regulating slippage errors [44,53]. Our more extensive investigation however does not support a simple linear relationship between EST-SSR abundance and genome size. For example, two gymnosperm genera such as *Gnetum* and *Zamia* have similar or even higher frequencies of SSRs than angiosperm genera such as *Citrus*, which has a smaller genome size. This suggests that other factors affecting genome evolution in both taxa need to be considered to explain EST-SSR abundance diversity in the plant kingdom.

EST-SSR abundance across EST fractions also differs between gymnosperm and angiosperms. In angiosperms, EST-SSRs are significantly more abundant in the 5′UTR fraction, while in gymnosperms there is on an average a higher abundance of EST-SSRs in the 3′UTR fraction. In angiosperms, a higher density of EST-SSRs in the UTR fractions has been reported previously [4,20,54,55]; while other studies support a higher abundance in the ORF fraction [44]. A higher EST-SSR abundance in the 5′UTR could be attributed to a regulatory role [56,57]. In *Cryptomeria*, a higher density of EST-SSRs in the ORF fraction has also been shown [37]. However, due to the limited number of studies performed on each EST fraction separately, a generalization on the relative abundance of SSRs across those fractions warrants further investigation.

### Motif size: while dimers dominate, hexamers are more common in the gymnosperm EST sequences

Our study reveals an overall higher abundance of dimers across all three genomic fractions (with six exceptions). In an EST-SSRs analysis that included lower and upper plant species, Victoria et al. [46] reported that trimers are more frequent in the majority of groups of higher plants; while individual studies in angiosperm trees have shown dimers as the most abundant motif in genera such as *Populus* [17,45] and *Eucalyptus* [16,34]. In *Quercus*, trimers were reported as the most abundant motif in the ORF fraction, while dimers were more frequent in the UTR fractions [20]. Trimers were the most common motif in *Citrus* according to some studies [19,27] whereas Palmieri et al. [28] described dimers as the most abundant motifs in the same genus. In gymnosperms, a higher abundance of EST-SSR dimers has previously reported in *Pinus*, *Picea*, and *Ginkgo* [5,24,45,46]; while Yan et al. [47] reported trimers as the most abundant in *Pinus*. Similarly, trimers were the most frequent in the ORF in *Pinus*, while dimers were the most common in the 3′UTR fraction [43]. In agreement with our study, increased representation of trimers in the ORF was shown before in *Cryptomeria* [37]. Trimers and hexamers were reported to be more common in the ORF compared to the UTRs in *Quercus* [20] and *Cryptomeria* [37]. Similarly, we also observe trimers and hexamers as common in both taxa with reference to ORF.

Our data shows that despite the fact that dimers are the most frequent repeats in majority of the genera in all the three genomic fractions, the proportion of dimers to trimers (dimers/trimers) decreases significantly in the ORF fraction. Predominance of trimers in the coding regions was reported previously in animals and plants [58]. ORF enrichment in trimers is expected considering that dimers alter the frameshift (i.e., nucleotide triplet or codon is the unit for translation), which should be avoided if the correct translation of the ORF into a protein should be maintained. Presence of SSR dimers in the ORF fraction can potentially affect gene amino acid sequences consequently altering their function due to frameshift mutations, while SSRs in the UTR fractions will affect transcription, translation or splicing of gene products [8]. Moreover, if the number of dimer repeats is divisible by three, it will result in the alternation of two amino acids (e.g., (AT)_6_: ATA-TAT-ATA-TAT: Ile-Tyr-Ile-Tyr), thus potentially leaving the reading frame un-altered, as previously suggested by Kantety et al. in cereal species [59].

### Dimer/Trimer nucleotide composition: AT-rich motifs are common in gymnosperms

Our study reveals a low abundance of AC/GT motif in all studied genera. Unlike as in mammals, the AC/GT motif is known to occur at low frequency in plants [4,60]. The difference between plants and mammals has been attributed to differences in methylation patterns. AC/GT abundance in animals was suggested as the result of transition of methylated C residue to T (CG/CG → AC/GT), while the absence of a C-hotspot in plants could have prevented the predominance of AC/GT repeats [4,60]. In agreement with previous works, the CG/CG motif (which creates CpG islands acting as regulatory elements through methylation) is almost absent in all our studied genera across all three genomic fractions. There is however an overall predominance of AG/CT (all three genomic fractions) and AAG/CTT (5′UTR and ORF) motifs in angiosperms, which are also target for methylation in plants [61]. In gymnosperms, AG/CT is also the most abundant motif in the 5′UTR and ORF fractions (with few genera where AT/AT is more abundant). In the 3′UTR regions, there is predominance of AT/AT (gymnosperms) and AAT/ATT (both taxa), which are not the target for methylation [62]. An increased content in A + T nucleotides in the 3′UTR fraction has been reported before in vertebrates [63], mammals [64], yeast [65] and *Arabidopsis* [4], which seems to be related to the UTR processing signal composition.

An overall predominance of AG/CT and AT/AT dimer motifs in EST sequences was supported by previous studies in angiosperms [20,34,47] and gymnosperms [5,46,47]. In angiosperms, AG/CT was reported as the most abundant in *Eucalyptus* [16,34,47], *Citrus* [26-28] and *Populus* [45,47,66]. In *Quercus*, AC/GT was shown as the most abundant dimer [20]. In agreement with an overall enrichment in AT/AT motif gymnosperms (specially in the 3′UTR fraction), other studies have also reported AT/AT as the most frequent dimer in *Pinus* [5,43,45-47], *Picea* [5,24,45] and *Ginkgo* [45]. Berube et al. [5] also demonstrate a similar finding with a higher abundance of AT/AT dimers in the 3′ sequenced ESTs in *Pinus* and *Picea*. The motif AG/CT was shown to be the most abundant in *Cycas* [45] and *Gnetum* [46]; the latter being also supported by our data. In *Cryptomeria*, AT/AT was shown to be the most abundant in the UTR fractions, while AG/CT was the most abundant in the ORF [37].

In agreement with our results, previous studies also support a higher abundance of the AAG/CTT motif in angiosperms. In gymnosperms, our study reveals predominance of the AAT/ATT motif in the 3′UTR fraction; moreover, trimer predominance in the other two fractions seems genus dependent. In angiosperms, AAG/CTT was ranked first in frequency in *Eucalyptus* [16,47], *Citrus* [26-28] and *Poplar* [45,47,66]. In *Eucalyptus*, other studies reported AGG/CCT [34] as the most abundant trimer motifs. In *Quercus*, AAT/ATT was shown to be the most common trimer motif [20]. In gymnosperms, AAT/ATT was shown to be the most abundant trimer in *Pinus* [45]. Other studies report AAG/CTT as the most common trimer in *Pinus* [43,47], *Picea* [24] and *Cycas* [45]. Also ACG/CGT was presented as the most abundant trimer in *Pinus* and *Picea* [5]. In *Cryptomeria*, our trimer motif dominance across the EST fractions corresponds with that reported by [37] (i.e., AGG, 5′UTR; AAG, ORF; AAT, 3′UTR).

## Conclusions

Our EST-SSR comparative analysis in eight angiosperm genera and seven gymnosperm genera has revealed interesting differential features among both taxa. While dimers dominate, hexamers are more common in the gymnosperm EST sequences than the angiosperms, and AT-rich motifs among the dimers are the most abundant in gymnosperms. These results provide the foundation for future comparative studies at the species level to unravel the evolutionary processes that control the SSR genesis and divergence between angiosperm and gymnosperm tree species.

## Methods

### Genomic resources and bioinformatics

Description of the EST resources analysed in this study is represented in Additional file 1: Table S1. ESTs from 40 species from eight genera in angiosperms and 17 species from seven genera in gymnosperms were considered for the EST-SSR analysis in this study. EST sequences of the selected species were retrieved from the dbEST database of the NCBI. The criterion for species selection, analysis and the results presented in this work was based on the availability of the sequence data in the EST databank. To remove redundancy, EST sequences were assembled into contigs and singlets, species-wise, using the sequence assembly program CAP3 with its default setting [67]. For each genus, the species-wise assembled contigs and singlets were pooled together and the sequence redundancy at genus level was removed using CD-HIT [68] with a cut off value of 90% (ensuring 90% sequence identity). The ORF detection is based on the same principle as the generic eukaryotic gene prediction program used for searching the coding regions from a given nucleotide sequence. Based on the coding potential profiles trained from Angiosperms (Arabidopsis) and Gymnosperms (Norway spruce) protein coding genes, we used AUGUSTUS [69] to distinguish the coding and the UTR regions, and the coding direction of a given transcript sequence. The main feature in detecting ORF on transcript sequence is that the ORF is located in an intron-less, single exon coding region. However, due to the unexpected higher coding potential in the UTR region, one transcript might contain more than one ORF. In such cases, we have selected the longest ORF as the true coding region and the adjacent nucleotide sequence as the UTR region. Thus the longest ORF was selected from each of the EST sequence from the genus-wise collection of sequences and the 5′UTR and 3′UTR fractions of the sequence were assigned based on the coordinate direction of the ORF. Three groups of sequences were thus created with reference to each genus, namely 5′UTR, ORF and 3′UTR. SSRLocatorI v.1 [70] was used to retrieve the SSR information at the genus level from each of the three groups derived. SSRLocator was used with the following settings, SSR repeat motifs and number of repeats shown respectively, dimer-10, trimer-7, tetramer-5, pentamer-4, hexamer-4, heptamer-3, octamer-3, nonamer-3, decamer-2. The space between compound SSRs was set to 100 bp. Thus repetitions that occurred in the adjacent regions lower than 100 bp, were considered as compound SSRs. These settings are in compliance with the search parameters for repetitive elements in class I (≥20 bp) described as more efficient molecular markers followed by Temnykh et al. [71]. Mononucleotide repeats can be difficult to accurately assay and are generally eliminated from the SSR analysis [45,72-74] and consequently these repeats were excluded from this study. Therefore, in this article we discuss the occurrence of microsatellites specific to 5′UTR, ORF or 3′UTR fractions of the ESTs. While recording the count of a particular repeat motif, circular permutations and/or reverse complements of each other were clustered together (e.g. AC = GT = CA = TG, ACG = CGA = GCA = TGC = GCT = CGT = AGC = TCG = CAG = GTC = TGC = GAC and AAC = ACA = CAA = TTG = TGT = GTT) [5]. We also screened for perfect and compound SSRs. Perfect SSRs are the repeat motifs that are simple tandem sequence, without any interruptions within the repeat (e.g. TATATATATATATATA or [TA]n); while a compound SSR consists of the sequence containing two adjacent distinct SSRs separated by none to any number of base pairs (e.g. TATATATATAGTGTGTGTGT or [TA]n-[GT]n).

### Statistical analysis

A non-parametric Tukey HSD test was carried to compare the means of EST-SSRs length between all categories. We carried out a 2 × 3 contingence *χ*2 test for heterogeneity of microsatellite counts (motif counts/total EST-fraction in Mbp) among the three EST genomic regions. Statistical analyses were all carried out using the R software package [75].

## Additional file

Additional file 1: Table S1. EST database size, number of nucleotides used for SSR analysis and counts of repeat motifs per Mbp in each fraction: (a) Angiosperms and (b) Gymnosperms. **Table S2** SSR motif complexity in: (a) Angiosperms and (b) Gymnosperms.

## Abbreviations

SSR: Simple sequence repeats
EST: Expressed sequence tags
UTR: Untranslated region
ORF: Open reading frame
Myr: Million years
TE: Transposable element
