# Supplementary material for: Comparative in silico analysis of EST-SSRs in angiosperm and gymnosperm tree genera
Source: BMC Plant Biol. 2014 Aug 21;14:220. doi: 10.1186/s12870-014-0220-8 (PMC4160553; doi:10.1186/s12870-014-0220-8)
Supplement: Additional file 1: Table S1. — EST database size, number of nucleotides used for SSR analysis and counts of repeat motifs per Mbp in each fraction: (a) Angiosperms and (b) Gymnosperms. Table S2 SSR motif complexity in: (a) Angiosperms and (b) Gymnosperms. [file 12870_2014_220_MOESM1_ESM.docx]

**Table1: EST database size, number of nucleotides used for SSR analysis and counts of repeat motifs per Mbp in each fraction: (a) Angiosperms and (b) Gymnosperms.**

**(a)**

| **Sr. No** | **Species** | **Number of ESTs from NCBI *** | **Total assembled nucleotides** | **Genus-wise non-redundant assembled sequences** | **Number of nucleotides analysed in the 5'UTR** | **Number of repeat motifs in 5'UTR** | **Counts per Mpb in 5'UTR** | **Number of nucleotides analysed in the ORF** | **Number of repeat motifs in ORF** | **Counts per Mpb in ORF** | **Number of nucleotides analysed in the 3'UTR** | **Number of repeat motifs in 3'UTR** | **Counts per Mpb in 3'UTR** |
| --- | --- | --- | --- | --- | --- | --- | --- | --- | --- | --- | --- | --- | --- |
| **Genus *Populus*** | | **221,720 (~3.0)** | 38,778,183 | 73,277 | 6,676,010 | 9,902 | 1,483 | 22,087,693 | 12,814 | 580 | 10,014,480 | 6,543 | 653 |
| 1 | *Populus* *deltoids* | 14,661 |  |  |  |  |  |  |  |  |  |  |  |
| 2 | *Populus* *euphratica* | 13,979 |  |  |  |  |  |  |  |  |  |  |  |
| 3 | *Populus* *nigra* (black poplar) | 51,361 |  |  |  |  |  |  |  |  |  |  |  |
| 4 | *Populus* *tomentiglandulosa* | 1,650 |  |  |  |  |  |  |  |  |  |  |  |
| 5 | *Populus* *tremula* (European aspen) | 37,313 |  |  |  |  |  |  |  |  |  |  |  |
| 6 | *Populus* *trichocarpa* (black cottonwood) | 89,943 |  |  |  |  |  |  |  |  |  |  |  |
| 7 | *Populus* *tremuloides* (quaking aspen) | 12,813 |  |  |  |  |  |  |  |  |  |  |  |
| **Genus *Eucalyptus*** | | **36,948  (2.2)** | 9,963,215 | 16,167 | 1,527,314 | 3,462 | 2267 | 5,903,767 | 7,367 | 1,248 | 2,532,134 | 1,615 | 638 |
| 1 | *Eucalyptus* *globulus* (blue gum) | 14,026 |  |  |  |  |  |  |  |  |  |  |  |
| 2 | *Eucalyptus* *grandis* (rose gum) | 1,950 |  |  |  |  |  |  |  |  |  |  |  |
| 3 | *Eucalyptus* *gunnii* (cider tree) | 19,841 |  |  |  |  |  |  |  |  |  |  |  |
| 4 | *Eucalyptus* *tereticornis* (red ironbark) | 1,131 |  |  |  |  |  |  |  |  |  |  |  |
| **Genus *Betula*** | | **5,688 (2.5)** | 735,936 | 2,315 | 106,106 | 149 | 1,404 | 507,108 | 453 | 893 | 122,722 | 116 | 945 |
| 1 | *Betula pendula* (European white birch) | 2,549 |  |  |  |  |  |  |  |  |  |  |  |
| 2 | *Betula platyphylla* (Asian white birch) | 3,139 |  |  |  |  |  |  |  |  |  |  |  |
| **Genus *Fagus*** | | **(2.5)** | 8,620,663 | 12,544 | 1,111,435 | 1,887 | 1,698 | 5,765,232 | 2,679 | 465 | 1,743,996 | 1,084 | 622 |
| 1 | *Fagus* *sylvatica* (European beech) | 31,309 |  |  |  |  |  |  |  |  |  |  |  |
| **Genus *Quercus*** | | 149,976 (3.4) | 27,897,186 | 43,500 | 4,283,827 | 11,734 | 2,739 | 16,713,255 | 15,853 | 949 | 6,900,104 | 7,650 | 1,109 |
| 1 | *Quercus* *mongolica* (Mongolian oak) | 3,385 |  |  |  |  |  |  |  |  |  |  |  |
| 2 | *Quercus* *petraea* (sessile oak) | 58,230 |  |  |  |  |  |  |  |  |  |  |  |
| 3 | *Quercus* *robur* (truffle oak) | 81,671 |  |  |  |  |  |  |  |  |  |  |  |
| 4 | *Quercus* *suber* (cork oak) | 6,690 |  |  |  |  |  |  |  |  |  |  |  |
| **Genus *Citrus*** | | **537,256 (2.3)** | 240,013,797 | 236,472 | 47,420,973 | 23,842 | 503 | 107,539,345 | 26,579 | 247 | 85,053,479 | 17,861 | 210 |
| 1 | *Citrus aurantiifolia* (lime) | 8,219 |  |  |  |  |  |  |  |  |  |  |  |
| 2 | *Citrus aurantium* (Seville orange) | 14,584 |  |  |  |  |  |  |  |  |  |  |  |
| 3 | *Citrus clementina* | 118,365 |  |  |  |  |  |  |  |  |  |  |  |
| 4 | *Citrus jambhiri* (rough lemon) | 989 |  |  |  |  |  |  |  |  |  |  |  |
| 5 | *Citrus latifolia* (Persian lime) | 8,756 |  |  |  |  |  |  |  |  |  |  |  |
| 6 | *Citrus limettioides* (sweet lime) | 8,188 |  |  |  |  |  |  |  |  |  |  |  |
| 7 | *Citrus limon* (lemon) | 1,505 |  |  |  |  |  |  |  |  |  |  |  |
| 8 | *Citrus limonia* (sharbati) | 11,045 |  |  |  |  |  |  |  |  |  |  |  |
| 9 | *Citrus macrophylla* (colo) | 1,929 |  |  |  |  |  |  |  |  |  |  |  |
| 10 | *Citrus medica* (citron) | 1,115 |  |  |  |  |  |  |  |  |  |  |  |
| 11 | *Citrus reshni* | 5,768 |  |  |  |  |  |  |  |  |  |  |  |
| 12 | *Citrus reticulata* (tangerine) | 55,980 |  |  |  |  |  |  |  |  |  |  |  |
| 13 | *Citrus sinensis* (apfelsine) | 213,830 |  |  |  |  |  |  |  |  |  |  |  |
| 14 | Citrus sunki | 5,216 |  |  |  |  |  |  |  |  |  |  |  |
| 15 | *Citrus trifoliata* (trifoliate orange) | 62,695 |  |  |  |  |  |  |  |  |  |  |  |
| 16 | *Citrus* *unshiu* (Satsuma orange) | 19,072 |  |  |  |  |  |  |  |  |  |  |  |
| **Genus *Prunus*** | | **104,402 (9.3)** | 6,460,876 | 11,193 | 1,044,150 | 8,317 | 7,965 | 3,889,628 | 12,014 | 3,089 | 1,527,098 | 6,929 | 4,537 |
| 1 | *Prunus* *armeniaca* (apricot) | 15,105 |  |  |  |  |  |  |  |  |  |  |  |
| 2 | *Prunus* *cerasus* (sour cherry) | 1,255 |  |  |  |  |  |  |  |  |  |  |  |
| 3 | *Prunus* *dulcis* (almond) | 3,864 |  |  |  |  |  |  |  |  |  |  |  |
| 4 | *Prunus* *mume* (ume) | 4,589 |  |  |  |  |  |  |  |  |  |  |  |
| 5 | *Prunus* *persica* (peach) | 79,589 |  |  |  |  |  |  |  |  |  |  |  |
| **Genus *Fraxinus*** | | **(1.9)** | 2,844,413 | 6,235 | 275,825 | 152 | 551 | 1,916,897 | 350 | 183 | 651,691 | 154 | 236 |
| 1 | *Fraxinus* *excelsior* (European ash) | 12,083 |  |  |  |  |  |  |  |  |  |  |  |

***** Ratio of total ESTs to clustered ESTs per genus is in between parenthesis.

**(b)**

| **Sr. No** | **Species** | **Number of ESTs from NCBI *** | **Total assembled nucleotides** | **Genus-wise non-redundant assembled sequences** | **Number of nucleotides analysed in the 5'UTR** | **Number of repeat motifs in 5'UTR** | **Counts per Mpb in 5'UTR** | **Number of nucleotides analysed in the ORF** | **Number of repeat motifs in ORF** | **Counts per Mpb in ORF** | **Number of nucleotides analysed in the 3'UTR** | **Number of repeat motifs in 3'UTR** | **Counts per Mpb in 3'UTR** |
| --- | --- | --- | --- | --- | --- | --- | --- | --- | --- | --- | --- | --- | --- |
| **Genus *Picea*** | | **514,092 (5.8)** | 67,685,052 | 88,120 | 11,880,217 | 2,937 | 247 | 35,698,156 | 7,360 | 206 | 20,106,679 | 5,029 | 250 |
| 1 | *Picea abies* (Norway spruce) | 14,345 |  |  |  |  |  |  |  |  |  |  |  |
| 2 | *Picea* *glauca* (white spruce) | 313,110 |  |  |  |  |  |  |  |  |  |  |  |
| 3 | *Picea* *sitchensis* (Sitka spruce) | 186,637 |  |  |  |  |  |  |  |  |  |  |  |
| **Genus *Pinus*** | | **452,484 (4.7)** | 64,010,078 | 95,397 | 10,700,169 | 2,310 | 216 | 35,039,173 | 6,464 | 184 | 18,270,736 | 3,413 | 187 |
| 1 | *Pinus* *banksiana* (jack pine) | 36,379 |  |  |  |  |  |  |  |  |  |  |  |
| 2 | *Pinus* *contorta* (lodgepole pine) | 40,483 |  |  |  |  |  |  |  |  |  |  |  |
| 3 | *Pinus* *densiflora* (Japanese red pine) | 3,316 |  |  |  |  |  |  |  |  |  |  |  |
| 4 | *Pinus* *pinaster* (maritime pine) | 34,261 |  |  |  |  |  |  |  |  |  |  |  |
| 5 | *Pinus* *radiata* (Monterey pine) | 8,717 |  |  |  |  |  |  |  |  |  |  |  |
| 6 | *Pinus* *sylvestris* (Scots pine) | 666 |  |  |  |  |  |  |  |  |  |  |  |
| 7 | *Pinus* *taeda* (loblolly pine) | 328,662 |  |  |  |  |  |  |  |  |  |  |  |
| **Genus *Cryptomeria*** | | **(2.5)** | 9,310,495 | 22,450 | 1,551,208 | 346 | 223 | 5,455,686 | 1,189 | 218 | 2,303,601 | 552 | 240 |
| 1 | *Cryptomeria* *japonica* (Japanese cedar) | 56,645 |  |  |  |  |  |  |  |  |  |  |  |
| **Genus *Gnetum*** | | **(1.9)** | 2,791,724 | 5,743 | 550,490 | 348 | 632 | 1,636,539 | 1,086 | 664 | 604,695 | 332 | 549 |
| 1 | *Gnetum* *gnemon* | 10,724 |  |  |  |  |  |  |  |  |  |  |  |
| **Genus *Cycas*** | | **(2.2)** | 5,522,585 | 10,153 | 869,283 | 150 | 173 | 3,374,440 | 366 | 108 | 1,278,862 | 510 | 399 |
| 1 | *Cycas* *rumphii* | 21,997 |  |  |  |  |  |  |  |  |  |  |  |
| **Genus *Zamia*** | | **20,677  (1.4)** | 4,645,568 | 14,504 | 825,241 | 503 | 610 | 2,578,366 | 1,807 | 701 | 1,241,961 | 911 | 734 |
| 1 | *Zamia* *fischeri* | 8,252 |  |  |  |  |  |  |  |  |  |  |  |
| 2 | *Zamia* *furfuracea* | 930 |  |  |  |  |  |  |  |  |  |  |  |
| 3 | *Zamia* *vazquezii* | 11,495 |  |  |  |  |  |  |  |  |  |  |  |
| **Genus *Ginkgo*** | | **(2.4)** | 4,694,213 | 9,127 | 598,607 | 231 | 386 | 2,789,129 | 585 | 210 | 1,306,477 | 704 | 539 |
| 1 | *Ginkgo* *biloba* (maidenhair tree) | 21,590 |  |  |  |  |  |  |  |  |  |  |  |

***** Ratio of total ESTs to clustered ESTs per genus is in between parenthesis.

**Table2: SSR motif complexity in: (a) Angiosperms and (b) Gymnosperms.**

**(a)**

| **Genus** | **Perfect SSR (%)** | | | **Compound SSR (%)** | | |
| --- | --- | --- | --- | --- | --- | --- |
|  | 5'UTR | ORF | 3'UTR | 5'UTR | ORF | 3'UTR |
| *Populus* | 96.8 | 95.8 | 96.9 | 3.2 | 4.2 | 3.1 |
| *Eucalyptus* | 96.2 | 92.6 | 95.0 | 3.8 | 7.4 | 5.0 |
| *Betula* | 94.1 | 100 | 100 | 5.9 | 0 | 0 |
| *Fagus* | 98 | 96.7 | 97.7 | 2 | 3.3 | 2.3 |
| *Quercus* | 96.4 | 96.6 | 98.0 | 3.6 | 3.4 | 2.0 |
| *Citrus* | 97.6 | 96.8 | 97.0 | 2.4 | 3.2 | 3.0 |
| *Prunus* | 96.1 | 95.2 | 95.6 | 3.9 | 4.8 | 4.4 |
| *Fraxinus* | 100 | 94.1 | 100 | 0 | 5.9 | 0 |

**(b)**

| **Genus** | **Perfect SSR (%)** | | | **Compound SSR (%)** | | |
| --- | --- | --- | --- | --- | --- | --- |
|  | 5'UTR | ORF | 3'UTR | 5'UTR | ORF | 3'UTR |
| *Picea* | 97.4 | 94.6 | 97.8 | 2.6 | 5.4 | 2.2 |
| *Pinus* | 97.6 | 94.9 | 95.2 | 2.4 | 5.1 | 4.8 |
| *Cryptomeria* | 97 | 99.4 | 97.3 | 3 | 0.6 | 2.7 |
| *Gnetum* | 96.1 | 94.1 | 100 | 3.9 | 5.9 | 0 |
| *Cycas* | 100 | 93.9 | 93.2 | 0 | 6.1 | 6.8 |
| *Zamia* | 90.7 | 95.1 | 97.6 | 9.3 | 4.9 | 2.4 |
| *Ginkgo* | 100 | 100 | 94.6 | 0 | 0 | 5.4 |
